# No one left behind: has the pursuit of FP2020’s 120 million additional users goal left some women behind?

**DOI:** 10.12688/gatesopenres.13339.1

**Published:** 2021-07-28

**Authors:** Shiza Farid, Jason Bremner, Emma Anderson

**Affiliations:** 1 FP2020, Washington, DC, 20006, USA

**Keywords:** global family planning, FP2020, FP2030, reproductive health, modern contraceptive prevalence

## Abstract

**Background: **An important question is whether the FP2020’s “120 million additional users” goal exacerbated inequities and led to a prioritization of populations within countries where substantial gains towards the goal could be made. We examine FP2020 country data and policies for signs of inequity in gains in modern contraceptive prevalence (MCP) and in the focus of family planning programs and policies.

**Methods: **We selected 11 countries (Bangladesh, Burundi, Ethiopia, Haiti, Malawi, Mali, Nepal, Pakistan, Senegal, Sierra Leone, Uganda, and Zimbabwe) to conduct a bivariate analysis. We evaluated if MCP growth had been equitable by assessing MCP between two surveys stratified by residence, levels of education, age groups, marital status, and wealth.

**Results: **In most countries, MCP increased among rural women and in seven African countries these gains were significant. In six countries, MCP gains were significant both among women with no education and in the lowest wealth group. MCP gains among young women aged 15-19 and 20-24 were seen in four African countries: Malawi, Senegal, Sierra Leone, and Uganda.

**Conclusions: **Our findings suggest that between two surveys since 2010 many countries saw MCP gains across different dimensions of equity and do not suggest a focus on expanded coverage at the expense of equity. As the family planning community begins to look ahead to the next partnership, this analysis can help inform the emerging FP2030 framework, which includes equity as a guiding principle.

## Introduction

In 2012 at the London Summit on Family Planning, key family planning partners came together to reinvigorate the family planning movement and accelerate progress toward universal access to family planning. The summit led to the creation of the Family Planning 2020 partnership (FP2020) with the aim of improving access to voluntary family planning information and services and enable 120 million additional women to use modern contraceptives in 69 focus countries by 2020. Many in the family planning community welcomed the new commitments of aid from donors, new commitments by country governments, and the push to bolster family planning as a development priority; however, there were also concerns about the focus on numbers of contraceptive users and a global goal ^1, 2^. Advocates pointed toward family planning’s past experiences of coercion and targets, warning that the FP2020 goal could lead to a focus on numbers and prioritization of easier to reach populations.

One outcome of this early concern was a call by the family planning community for a clear focus on rights and empowerment as guiding principles of the FP2020 partnership ^3^. A Rights and Empowerment Working Group was established by FP2020 to advance rights-based family planning, and the group provided early guidance to FP2020 and the family planning community. The FP2020 partnership now recognizes 10 rights and empowerment principles of family planning, including agency and autonomy; availability; accessibility; acceptability; quality; empowerment; equity and non-discrimination; informed choice; transparency and accountability; and voice and participation ^4^. Over the last eight years of the partnership, FP2020 worked with partners to ensure that these rights principles are understood by family planning decision-makers, and incorporated into new commitments and family planning-costed implementation plans.

But monitoring whether these rights principles are adhered to has remained a measurement challenge that FP2020 and partners are still working on. Thus, one outstanding question as FP2020 begins to look toward the Family Planning 2030 (FP2030) partnership, is whether the FP2020 goal exacerbated inequities and led to a prioritization of populations within countries where substantial gains towards the 120 million goal could be made. Ultimately, we aim to examine whether the effort to accelerate progress and expand contraceptive use has left some women behind. This question is particularly relevant in the context of a family planning partnership that supports the Sustainable Development Goals and the pledge that no one will be left behind ^5^. To assess this question, we examined FP2020 country progress data for signs of inequity in the focus of programs or gains in modern contraceptive prevalence (MCP), in FP2020 focus countries since 2012. This research is critical for informing the FP2030 partnership (which builds on the work of FP2020), as family planning policy makers begin to turn their attention to new commitments.

### Family planning progress and assessing inequity

FP2020’s annual reporting of contraceptive use has been facilitated by the adoption of statistical models that allow the estimation of a number of key family planning indicators, including MCP, using all available surveys and country health management information system statistics ^6^. These data indicate that as of July 2020, there were over 320 million total users of modern methods of contraception in the 69 FP2020 focus countries, i.e. 60 million additional users of modern contraception as compared to 2012. Since 2012, 26 countries have each gained more than 500,000 additional users of modern methods. Among these countries, 14 have seen the number of additional users grow by more than one million women and girls. While the pace of progress has been far short of the acceleration needed to achieve the FP2020 goal of 120 million additional users by 2020, there has been a clear focus by countries on increasing family planning coverage. As part of their FP2020 commitments, 45 countries established goals of increasing contraceptive prevalence through voluntary family planning programs. Almost all FP2020 countries addressed inequity in their FP2020 commitments as well, principally through commitments to improve access for adolescents and youth (41 countries), but also through efforts to address wealth and geographic inequities (13 countries). Progress has varied across regions and countries, but many countries across sub-Saharan and West Africa have seen rapid annual gains in modern contraceptive prevalence since 2012, most notably Mozambique at 2.7 percentage points per year, Burkina Faso at 1.4 percentage points, Malawi, and Sierra Leone at 1.3 percentage points, and Senegal at 1.2 percentage points.

Many researchers have addressed how to evaluate equitable access to public health programs, including family planning ^7– 11^. One study across various areas of health concluded residence, race/ethnicity, occupation, gender/sex, religion, education, socioeconomic status, and social status are key sociodemographic factors that can elucidate inequities ^12, 13^. A recent special issue analyzed inequalities in coverage of reproductive, maternal, newborn, and child health, and illustrated the wide inequality in reproductive health coverage in many countries across several dimensions, including wealth, age, and geography ^14^. These analyses, however, did not examine changes in inequity over time, and the most recent examination of contraceptive prevalence was an examination of trends through the Millennium Development Goals era, relying only on surveys through 2013.

While there are multiple dimensions to equity, most researchers agree that wealth is essential to assess disparities in contraceptive use. Recently published literature on FP2020 countries suggests poverty among married women declined over time while modern contraceptive use increased ^15^. Furthermore, an analysis of 46 countries completed using data from many FP2020 countries (though outside the FP2020 period) from national surveys from 1990–2013, found that the contraceptive use gap between the poorest and the richest has narrowed and modern methods account for nearly all the increase in contraceptive use ^16^.

In addition to examining differences related to wealth, in this analysis, we evaluate whether the growth in MCP has been equitable across multiple demographic characteristics, including residence (rural and urban), level of education (no education, primary, secondary, higher), age groups (15–19, 20–24, 25–29, 30–34, 35, 39, 40–44, 45–49), and marital status (married, all women, and unmarried sexually active ^i^).

## Methods

We examined data availability across the 69 FP2020 countries and selected 11 countries (Bangladesh, Burundi, Ethiopia, Haiti, Malawi, Mali, Nepal, Pakistan, Senegal, Sierra Leone, Uganda, and Zimbabwe), as they had data from two surveys ^ii^ of the same survey type (Demographic and Health Surveys [DHS] or Multiple Indicators Cluster Surveys [MICS]) between 2010 and 2019, and were an FP2020 commitment-making country. Countries that did not meet these criteria were excluded ^iii^. While FP2020 annual progress reporting relies on national-level modeled estimates of MCP, in this analysis we focused on survey data because models do not yet produce estimates for the majority of the dimensions of inequity examined in this paper. We included surveys from 2010 so we could complete the analysis for a larger pool of countries and assess progress during the years of the FP2020 partnership. The average number of years between two surveys was more than four. We also focused exclusively on FP2020 commitment-making countries because these countries had explicitly pledged to increasing contraceptive use.

We evaluated whether MCP growth had been equitable by assessing MCP between two surveys stratified by residence (rural, urban), levels of education (no education, primary, secondary, higher), age groups (15–19, 20–24, 25–29, 30–34, 35, 39, 40–44, 45–49), marital status (married, all women, unmarried sexually active), and wealth (poorest, poorer, middle, richer, richest). In this paper, we present MCP by levels of the abovementioned dimensions: wealth, residence, education, age, marital status, and sexual activity. We conducted a bivariate analysis where MCP estimates for married women were stratified by each of the socio-demographic dimensions, and weighted using survey-specific weights; confidence intervals were also calculated. Estimates of MCP by age and for unmarried, sexually active women were calculated in the same manner but were for all women in the survey. MCP gains were considered significant if the 95% confidence intervals for MCP stratified by the above-mentioned different socio-demographic dimensions did not overlap between surveys. Though this approach to testing the differences in means using hypothesis testing is more conservative, this approach can be easily interpreted by a wider audience. The MCP gains were considered equitable if larger or equivalent gains were seen in comparatively more disadvantaged populations. In this analysis, we qualify disadvantaged populations as those that are rural, have no education, aged 15–19, unmarried and sexually active in the last 30 days, or the poorest (or poorer group).

To assess MCP change by wealth, we did not use the DHS or MICS calculated wealth quintiles as those should not be compared across surveys nor time ^17^. Instead, we constructed five wealth groups based on Global Data Lab’s International Wealth Index (IWI) guidance using household assets; the reconstruction of wealth groups allowed for cross-country and across-time comparisons ^18^. Global Data Lab’s methods uses factor-loadings produced from a principal components analysis using data from over two million households across 97 countries. If, based on the IWI, 50% of a country’s women reside in households with less than 50% of the assets, these women live in households that are among the poorer households globally.

We also evaluated whether the number of women in each of the socio-demographic dimensions changed between surveys. For example, did fewer women have “no education” in the latest survey compared to the older survey? Or, did households in which women reside gain wealth over time? These findings can also help contextualize if MCP gains were made in more advantaged socio-demographic groups at the expense of most of the population (provided that the most advantaged group in any country will make up a smaller proportion of the total population).

For MICS surveys from Sierra Leone, all data cleaning was completed in RStudio (version 4.0.2) since MICS surveys are saved as SPSS file and Stata 15 does not support SPSS files. For all DHS surveys, Stata 15 was used to complete the analysis.

### Ethical considerations

The DHS Program procedures and questionnaires have been approved by ICF Institutional Review Board. Furthermore, additional approval is obtained by the IRB in the country of the survey. According to the DHS Program, all surveys are in compliance with the Department of Health and Human Services policies for protection of human subjects and the IRB in the country of the survey ensures the questionnaire is in compliance with the country’s laws and norms. Moreover, before each interview is conducted participants are read a consent statement – the participant can accept or decline to participate. The consent emphasizes that participation is voluntary. Additional information on DHS Program’s ethical standards can be found here: https://dhsprogram.com/methodology/Protecting-the-Privacy-of-DHS-Survey-Respondents.cfm.

The MICS Program under UNICEF similarly follows strict ethical guidelines. According to MICS, during the planning and designing stage of the survey, a governing structure is established. This governing structure is responsible for the formation of the steering and technical committees that are responsible for the implementation of the survey. The steering and technical committees will include focal points for ethical review submission and process. Cultural norms will also be used to adapt questionnaires as needed. Informed consent of all interviewees is required, and participation is voluntary.

## Results

**Figure 1.**
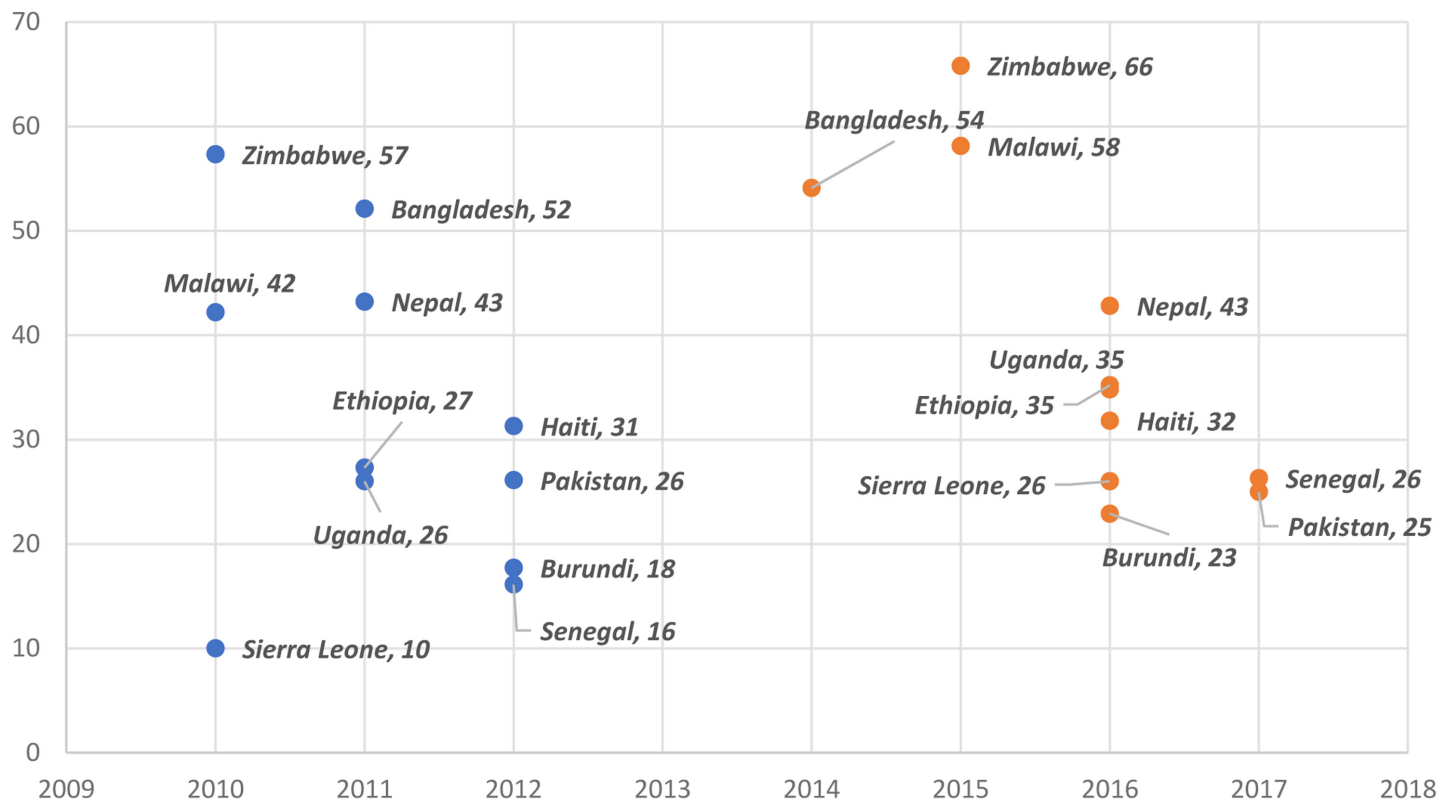
Burundi, Ethiopia, Malawi, Senegal, Sierra Leone, Uganda, and Zimbabwe had significant increases in modern contraceptive prevalence (MCP). Blue represents older survey and orange represents latest survey.

Overall, Figure 1 illustrates that MCP increased in nine out of the 11 countries, though the increases were only significant in seven African countries: Burundi, Ethiopia, Malawi, Senegal, Sierra Leone, Uganda, and Zimbabwe.

### Wealth

Table 1 shows the changes in the size of wealth groups between the two surveys. In all 11 countries overall household wealth increased between the two surveys – i.e. the number of households in the poorest (those with 20% of the assets or less) or poorer (those with between 21–40% of assets) groups decreased. In Sierra Leone, every change in wealth group was significant and households accumulated more assets (i.e. more wealth) between the two surveys (2011–2016).

**Table 1.** How the size of wealth groups changed between surveys (only significant findings at 95% confidence are included). *A downward arrow suggests wealth groups size decreased and households moved out of those groups and into another group*.

	Poorest	Poorer	Middle	Richer	Richest
**Bangladesh**	**↓**			**↑**	
**Burundi**	**↓**	**↑**	**↑**		
**Ethiopia**					
**Haiti**					
**Malawi**	**↓**	**↑**	**↑**	**↑**	
**Nepal**	**↓**		**↑**	**↑**	
**Pakistan**		**↓**		**↑**	
**Senegal**	**↓**				**↑**
**Sierra Leone**	**↓**	**↓**	**↑**	**↑**	**↑**
**Uganda**		**↓**	**↑**	**↑**	
**Zimbabwe**					

**Figure 2.**
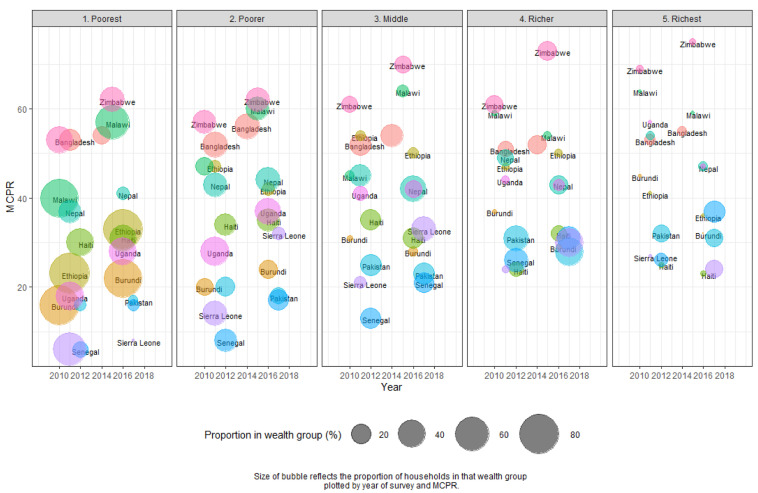
Modern contraceptive prevalence rate (MCPR) by wealth group between two surveys.

Table 2 shows the changes in MCP between wealth groups between two surveys. Figure 2 illustrates the changes in size of wealth group and MCP by survey. In every country, MCP increased in the lowest wealth group (those with 20% of the assets or less). These findings were significant in six countries – Burundi, Ethiopia, Malawi, Senegal, Uganda, and Zimbabwe. In Malawi, Senegal, Uganda, and Zimbabwe, the increase was around 10%; and in Malawi, the increase among the poorest was 16%.

**Table 2.** MCPR by wealth groups by country with significant MCPR increase denoted by * next to most recent survey estimate confidence interval.

Country	Survey	Poorest	Poorer	Middle	Richer	Richest
Bangladesh	2014 DHS	54 [51-58]	56 [54-58]	54 [51-56]	52 [49-55]	55 [50-59]
Bangladesh	2011 DHS	53 [51-55]	52 [50-54]	52 [50-54]	51 [48-53]	53 [49-57]
Burundi	2016-17 DHS	22 [21-23] *	24 [22-27]	28 [24-33]	29 [21-38]	32 [22-45]
Burundi	2010 DHS	16 [15-18]	20 [17-24]	31 [22-42]	37 [29-45]	45 [31-60]
Ethiopia	2016 DHS	33 [30-35] *	42 [37-47]	50 [43-57]	50 [43-56]	36 [27-45]
Ethiopia	2011 DHS	23 [21-26]	47 [41-53]	54 [46-61]	47 [39-55]	41 [33-49]
Haiti	2016-17 DHS	31 [28-33]	35 [31-37]	31 [31-39]	32 [21-28]	23 [17-34]
Haiti	2012 DHS	30 [15-18]	34 [17-24]	35 [22-42]	24 [29-45]	25 [31-60]
Malawi	2015-16 DHS	57 [55-58] *	60 [58-62] *	64 [60-68] *	54 [48-60]	59 [53-66]
Malawi	2010 DHS	40 [39-42]	47 [45-50]	45 [40-50]	59 [52-67]	64 [52-75]
Nepal	2016 DHS	41 [37-46]	44 [41-46]	42 [40-44]	43 [40-46]	47 [41-53]
Nepal	2011 DHS	37 [34-41]	43 [40-46]	45 [41-49]	49 [45-53]	54 [46-62]
Pakistan	2017-18 DHS	17 [12-22]	18 [18-23]	23 [23-28]	28 [29-33]	31 [30-35]
Pakistan	2012-13 DHS	16 [12-23]	20 [16-21]	25 [20-26]	31 [26-30]	32 [28-34]
Senegal	2017 DHS	16 [14-19] *	17 [16-19] *	21 [19-24] *	31 [28-34]	37 [34-41] *
Senegal	2012 DHS	6 [4-10]	8 [7-10]	13 [11-16]	26 [21-31]	26 [20-32]
Sierra Leone	2017 MICS	8 [1-40]	32 [25-41] *	33 [27-39] *	30 [25-35]	24 [18-31]
Sierra Leone	2011 MICS	6 [5-7]	14 [12-16]	21 [17-25]	24 [19-30]	27 [17-40]
Uganda	2016 DHS	28 [26-30] *	37 [35-39] *	42 [39-45]	43 [39-47]	47 [39-55]
Uganda	2011 DHS	18 [16-20]	28 [26-30]	41 [36-46]	44 [34-56]	57 [45-69]
Zimbabwe	2015 DHS	62 [60-65] *	62 [59-66]	70 [66-74] *	73 [70-76] *	75 [68-81]
Zimbabwe	2010-11 DHS	53 [51-56]	57 [53-61]	61 [57-65]	61 [58-65]	69 [59-77]

MCPR, modern contraceptive prevalence rate; DHS, Demographic and Health Survey; MICS, Multiple Indicator Cluster Surveys.

In all countries except Bangladesh, women with average wealth (those in the middle of the five wealth groups) had higher modern method use than in the lowest wealth groups. However, the gap in modern method use between those in the middle wealth group and the lowest wealth group declined in most countries between surveys. Growth in MCP was more uneven when comparing growth between surveys in higher wealth groups. All but three countries saw declines in MCP in the highest wealth groups, although these findings were not significant given the lower sample sizes in the richest groups per country. Conversely, in Senegal, there was a significant increase in MCP among the richest, which corresponds with a large significant increase in the number of people in the richest wealth group. See Table 2 below for additional comparisons.

### Residence

**Figure 3.**
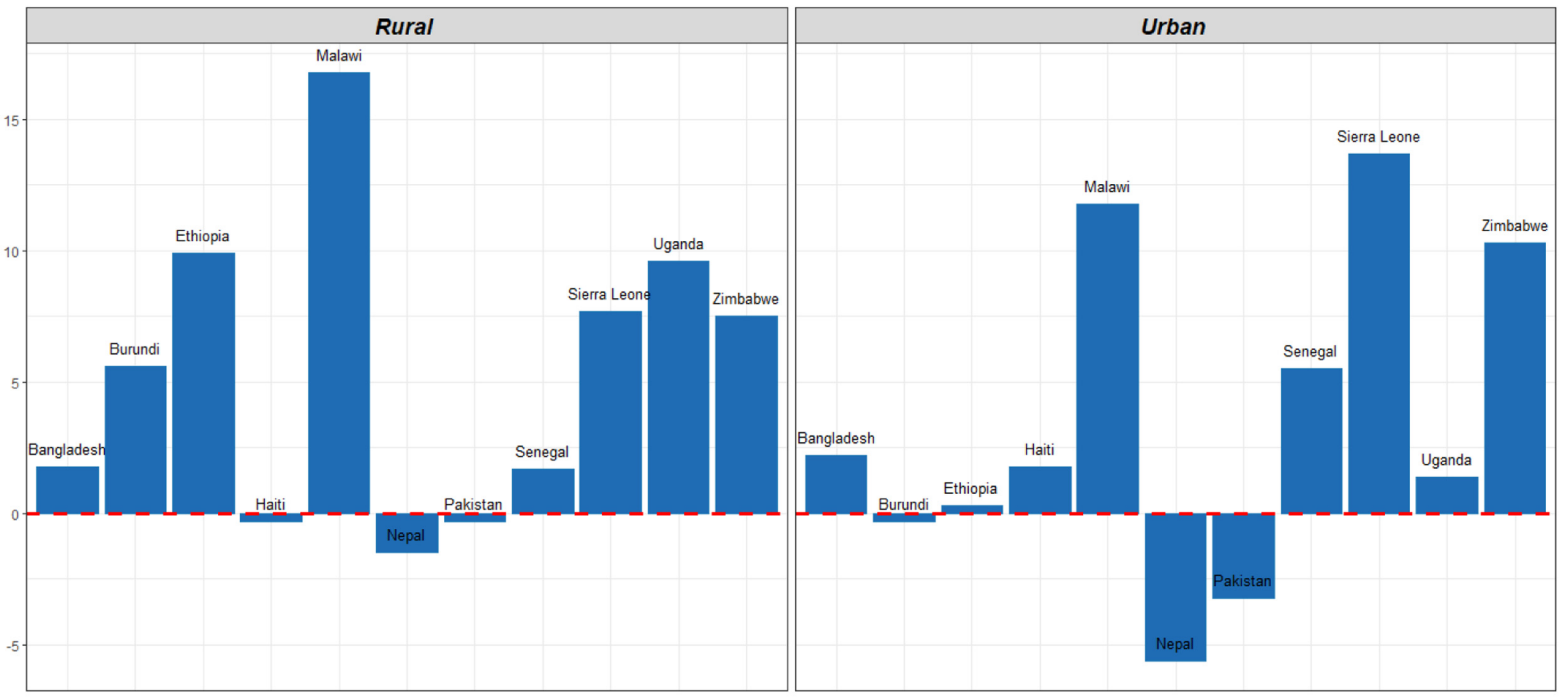
Change in modern contraceptive prevalence rate (MCPR) by residence between two surveys.

Table 3 shows the MCP for countries disaggregated by residence. In every country in our analysis, modern contraceptive use was higher in urban areas than rural areas. Modern contraceptive use in rural and urban areas increased in most countries. Increases were significant in seven countries– Burundi, Ethiopia, and Uganda in rural settings, and in Malawi, Senegal, Sierra Leone, and Zimbabwe in both rural and urban settings. In three countries, MCP declined among rural women between surveys: Haiti, Nepal, and Pakistan, though the decline was not significant. In both Pakistan and Nepal, MCP among urban women also declined between surveys. The largest gain among rural women was seen in Malawi where MCP increased by 17% between 2010 and 2016. Figure 3 illustrates the change in MCP between two surveys.

**Table 3.** MCPR by residence by country with significant MCPR increase denoted by * next to most recent survey estimate confidence interval.

Country	Survey	Urban	Rural
Bangladesh	2014 DHS	56 [54-58]	53 [52-55]
Bangladesh	2011 DHS	54 [52-56]	51 [50-53]
Burundi	2016-17 DHS	29 [25-32]	22 [21-24] *
Burundi	2010 DHS	29 [25-33]	17 [15-18]
Ethiopia	2016 DHS	50 [46-54]	32 [30-35] *
Ethiopia	2011 DHS	50 [46-53]	23 [20-25]
Haiti	2016-17 DHS	33 [30-36]	31 [29-33]
Haiti	2012 DHS	31 [29-34]	31 [29-34]
Malawi	2015-16 DHS	61 [59-64] *	58 [56-59] *
Malawi	2010 DHS	50 [46-53]	41 [39-42]
Nepal	2016 DHS	44 [42-46]	41 [38-43]
Nepal	2011 DHS	50 [47-53]	42 [40-45]
Pakistan	2017-18 DHS	29 [27-31]	23 [21-25]
Pakistan	2012-13 DHS	32 [30-35]	23 [22-25]
Senegal	2017 DHS	37 [34-39] *	19 [17-21] *
Senegal	2012 DHS	27 [24-31]	9 [8-11]
Sierra Leone	2017 MICS	30 [28-32] *	16 [14-17] *
Sierra Leone	2011 MICS	16 [13-19]	8 [7-9]
Uganda	2016 DHS	41 [38-43]	33 [31-35] *
Uganda	2011 DHS	39 [36-43]	23 [22-25]
Zimbabwe	2015 DHS	71 [68-73] *	63 [61-66] *
Zimbabwe	2010-11 DHS	60 [57-64]	56 [54-58]

MCPR, modern contraceptive prevalence rate; DHS, Demographic and Health Survey; MICS, Multiple Indicator Cluster Surveys.

### Education

**Figure 4.**
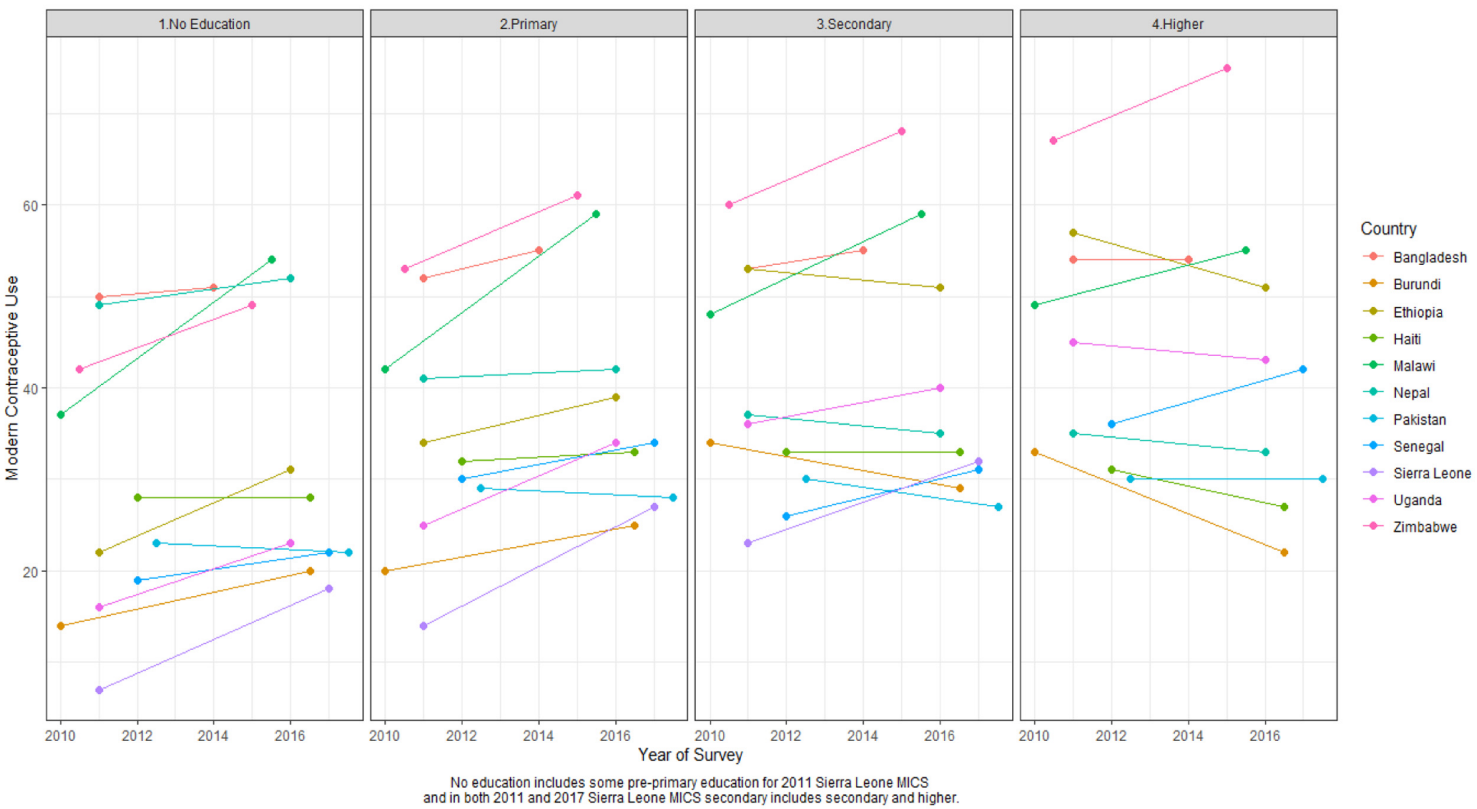
Modern contraceptive prevalence rate (MCPR) by education status between two surveys MICS, Multiple Indicator Cluster Surveys.

Table 4 shows the MCP stratified by education status between two surveys and Figure 4 illustrates the same information visually. In six of the 11 countries there were significant changes in MCP between surveys related to education: Burundi, Ethiopia, Malawi, Senegal, Sierra Leone, and Uganda. In all six countries, gains in modern contraceptive use were made among women with no education. In five of six countries– Burundi, Malawi, Senegal, Sierra Leone, and Uganda, significant MCP gains were also made among women with primary education. In Malawi and Sierra Leone, MCP gains among women with secondary education were also significant. The proportion of women with no education declined between two surveys in four of the six countries (and not in Ethiopia or Sierra Leone). Malawi had the fastest MCP growth where it grew by more than 16% among women with no education. Modern use remained stagnant (i.e. change was not statistically significant) among women in the highest education group in most countries except in Sierra Leone where it increased. Note, in Sierra Leone, the highest education category includes secondary and higher education, while in other countries those categories are separate. Among women in the highest education group, traditional method use increased in Burundi and Uganda by more than one percentage point. Further analysis is needed to discern if women in the highest education group in Burundi and Uganda discontinued a modern method for a traditional method or have been long-term traditional method users.

**Table 4.** MCPR by education by country with significant MCPR increase denoted by * next to most recent survey estimate confidence interval.

Country	Survey	No education	Primary	Secondary	Higher
Bangladesh	2014 DHS	51 [48-53]	55 [53-57]	55 [54-57]	54 [51-57]
Bangladesh	2011 DHS	50 [48-52]	52 [51-54]	53 [51-54]	54 [51-57]
Burundi	2016-17 DHS	20 [19-22] *	25 [23-27] *	29 [26-32]	22 [15-31]
Burundi	2010 DHS	14 [13-16]	20 [18-22]	34 [29-39]	33 [23-46]
Ethiopia	2016 DHS	31 [28-34] *	39 [35-43]	51 [45-56]	51 [44-57]
Ethiopia	2011 DHS	22 [19-24]	34 [31-37]	53 [46-61]	57 [50-64]
Haiti	2016-17 DHS	28 [25-31]	33 [31-36]	33 [31-36]	27 [21-34]
Haiti	2012 DHS	28 [25-31]	32 [30-34]	33 [31-36]	31 [25-38]
Malawi	2015-16 DHS	54 [51-56] *	59 [58-60] *	59 [56-61] *	55 [49-62]
Malawi	2010 DHS	37 [35-40]	42 [41-44]	48 [46-51]	49 [39-60]
Nepal	2016 DHS	52 [49-54]	42 [39-45]	34 [32-37]	33 [31-36]
Nepal	2011 DHS	49 [46-52]	41 [37-44]	37 [34-40]	35 [30-40]
Pakistan	2017-18 DHS	23 [22-25]	29 [26-32]	30 [28-33]	30 [27-33]
Pakistan	2012-13 DHS	22 [20-23]	28 [25-32]	27 [25-30]	30 [27-34]
Senegal	2017 DHS	22 [20-23] *	34 [31-36] *	31 [27-34]	42 [33-51]
Senegal	2012 DHS	12 [10-13]	25 [21-28]	27 [21-34]	49 [30-68]
Sierra Leone ^/ ^^	2017 MICS	18 [18-20] *	27 [24-30] *	32 [29-36] *	
Sierra Leone ^^	2011 MICS	7 [6-8]	14 [12-17]	23 [21-26]	
Uganda	2016 DHS	23 [20-26] *	34 [33-36] *	40 [38-43]	43 [39-47]
Uganda	2011 DHS	16 [13-19]	25 [23-27]	36 [33-40]	45 [38-51]
Zimbabwe	2015 DHS	49 [37-62]	61 [57-64]	68 [66-69]	75 [70-80]
Zimbabwe	2010-11 DHS	42 [34-51]	53 [50-56]	60 [58-62]	67 [59-73]

^ No education includes some pre-primary education and ^^ secondary includes secondary and higher MCPR, modern contraceptive prevalence rate; DHS, Demographic and Health Survey; MICS, Multiple Indicator Cluster Surveys.

### Age

Table 5 provides MCP by age group for all the countries. When comparing changes in MCP in individual age groups between surveys, we found that any statistically significant MCP increases were consistent for both married women and all women ^iv^. When comparing MCP increase by age groups between two surveys, we found no significance for four countries: Bangladesh, Haiti, Nepal, and Pakistan; significance in some age groups for Burundi, Ethiopia, and Zimbabwe; and significance in all age ranges for Malawi and Senegal. In Uganda and Sierra Leone, all age groups (except 45–49) saw a statistically significant increase in MCP use. The age group 20–24 showed significance across three of the countries, Burundi, Malawi, and Senegal, while Ethiopia showed significance for 25–29 years old and 40–44 years old. In Zimbabwe, the only age groups not showing significant change were the youngest two groups: 15–19 and 20–24-year-olds.

**Table 5.** Age groups by country with significant MCPR increase denoted by * next to most recent survey estimate confidence interval.

Country	Survey	15–19	20–24	25–29	30–34	35–39	40–44	45–49	Total
Bangladesh	2014 DHS	46 [43–48]	54 [51–56]	60 [58–62]	62 [60–64]	56 [54–59]	41 [38–43]	21 [19–24]	51 [50–52]
Bangladesh	2011 DHS	41 [39–44]	52 [50–54]	58 [56–60]	58 [56–60]	53 [50–55]	41 [39–44]	25 [23–28]	49 [48–50]
Burundi	2016–17 DHS	3 [2–3]	15 [14–17] *	20 [19–22]	22 [20–24]	21 [19–23]	16 [14–19]	10 [8–12]	15 [14–15] *
Burundi	2012 DHS	1 [1–2]	12 [10–14]	18 [16–20]	18 [15–21]	16 [14–19]	12 [10–14]	7 [5–10]	11 [10–12]
Ethiopia	2016 DHS	7 [6–9]	26 [23–29]	35 [32–38] *	35 [31–38]	29 [26–33]	28 [24–32] *	16 [13–21]	25 [23–27] *
Ethiopia	2011 DHS	5 [4–6]	22 [19–25]	26 [23–28]	28 [25–31]	26 [23–29]	19 [15–23]	10 [8–13]	19 [17–20]
Haiti	2016–17 DHS	8 [7–9]	25 [23–27]	31 [29–34]	30 [27–33]	29 [26–32]	24 [21–27]	13 [11–15]	22 [21–23]
Haiti	2012 DHS	8 [7–10]	23 [21–26]	31 [29–34]	31 [28–34]	28 [24–31]	22 [20–25]	14 [11–16]	22 [20–23]
Malawi	2015–16 DHS	15 [14–17] *	46 [44–48] *	57 [54–59] *	60 [58–62] *	59 [57–62] *	53 [50–56] *	43 [40–47] *	45 [44–46] *
Malawi	2010 DHS	9 [8–10]	33 [31–35]	42 [40–44]	42 [40–45]	44 [42–47]	40 [37–43]	33 [30–36]	33 [32–34]
Nepal	2016 DHS	4 [3–5]	18 [16–20]	34 [31–37]	46 [42–49]	56 [53–59]	55 [52–58]	52 [48–56]	33 [32–35]
Nepal	2011 DHS	4 [3–5]	18 [16–21]	36 [33–40]	50 [47–54]	57 [54–61]	56 [53–60]	44 [40–49]	33 [32–35]
Pakistan	2017–18 DHS	6 [4–9]	13 [11–16]	21 [18–23]	29 [27–32]	31 [29–34]	34 [31–37]	24 [22–29]	24 [23–26]
Pakistan	2012–13 DHS	7 [4–10]	15 [13–17]	21 [19–23]	31 [28–34]	35 [32–38]	31 [27–34]	24 [21–27]	25 [24–26]
Senegal	2017 DHS	3 [2–3] *	14 [12–15] *	23 [21–25] *	29 [26–31] *	31 [28–35] *	29 [26–32] *	21 [18–24] *	19 [18–20] *
Senegal	2012 DHS	1 [1–2]	8 [7–10]	16 [14–19]	16 [13–20]	22 [18–27]	18 [14–23]	9 [6–12]	11 [10–13]
Sierra Leone	2016 MICS	20 [19–22] *	33 [31–36] *	31 [28–33] *	28 [26–30] *	25 [23–27] *	19 [17–21] *	11 [9–13]	26 [24–27] *
Sierra Leone	2010 MICS	5 [3–7]	9 [7–11]	9 [7–11]	12 [10–14]	14 [12–16]	11 [9–14]	7 [5–10]	10 [9–11]
Uganda	2016 DHS	9 [8–11] *	28 [26–30] *	37 [35–40] *	37 [34–39] *	36 [34–39] *	34 [31–37] *	19 [17–22]	27 [27–28] *
Uganda	2011 DHS	6 [5–7]	20 [18–22]	28 [25–31]	30 [27–34]	30 [27–34]	27 [23–31]	14 [11–18]	21 [20–22]
Zimbabwe	2015 DHS	12 [11–14]	49 [46–52]	62 [59–65] *	66 [64–68] *	64 [61–67] *	56 [53–60] *	42 [37–47] *	48 [47–49] *
Zimbabwe	2010–11 DHS	10 [9–12]	44 [41–47]	55 [52–58]	55 [52–58]	52 [49–55]	45 [41–50]	31 [28–35]	41 [39–42]

This table represents an all women sample and an ever-married women sample in Pakistan and Bangladesh. MCPR, modern contraceptive prevalence rate; DHS, Demographic and Health Survey; MICS, Multiple Indicator Cluster Surveys.

### Unmarried sexually active ^v^

We also assessed whether there had been an increase in MCP among unmarried sexually active women. Typically, the sample of women that are unmarried and sexually active in the last 30 days is small which makes testing for significant changes in MCP challenging (since a larger sample increases confidence in our estimates and the confidence intervals are tighter allowing for detectable changes). While none of the findings were significant, we did see an increase in modern method use in Malawi and Zimbabwe.

## Discussion

A criticism of the “120 million additional users” goal of the FP2020 partnership had been that countries and partners might prioritize easier to reach populations (e.g. with technical or political support from the FP2020 partnership) to meet this target, which exacerbates inequities. Overall, our findings suggest that between two surveys carried out since 2010, many countries saw MCP gains across different dimensions of equity, including residence, education, wealth, age, and marital status and do not suggest a focus on expanded coverage at the expense of equity. In most countries, MCP increased among rural women, and these gains were significant in seven African countries. In six countries, MCP gains were significant both among women with no education and in the lowest wealth group. MCP gains among young women aged 20–24 were seen in four African countries: Malawi, Senegal, Sierra Leone, and Uganda. In these four countries, MCP also significantly increased among young women aged 15–19. While we could not detect significant MCP changes among unmarried sexually active women, the country-specific trends indicate MCP is increasing among these women. All of the significant gains in MCP were made in African countries, and specifically Malawi, who experienced the most expansive growth across different dimensions of equity.

Another possible criticism of the “120 million additional users” goal could be that the target would lead to a focus on more populous countries, but most of the gains in MCP were in African countries with populations far smaller than the those of several of the Asian countries in our sample. Both Pakistan and Bangladesh rank in the top 10 most populous countries in the world. Yet, our analysis found that despite having larger populations, neither of these countries experienced the rapid MCP growth of several of the smaller countries.

Compared to these Asian countries, most of the African countries in our analysis started at a lower MCP and thus had greater potential for increased modern contraceptive use. However, it is worth noting Pakistan had more room to grow than Malawi, who experienced the most overall MCP growth and saw growth across different equity dimensions. Additionally, given all countries in our analysis are still experiencing population growth and thus an increasing population of women of reproductive age, even countries such as Bangladesh, Nepal, and Pakistan, that did not have significant levels of MCP growth, were providing contraceptive services to a greater number of women of reproductive age without experiencing declines in equity.

Given that countries could either experience (a) rapid MCP growth (because they started at a lower MCP in the middle of the S-curve growth pattern ^19^) or (b) sustained modern contraception users (meaning they continue to add more users of modern contraception due to population growth and a slower increase in MCP), supportive country-specific policies and commitments that focused on expanding services and improving equity could be at the nexus of increasing and/or retaining modern contraceptive users without exacerbating inequities. In order to sustain its high MCP, government and development partners in Bangladesh successfully focused on geographic inequities; both the Sylhet and Chittagong divisions had the lowest MCP in 2011 and saw significant increase in the 2014 DHS (2 years after Bangladesh committed to improving family planning services in these two divisions). Similarly, Malawi successfully prioritized young people in its commitments and policies. In fact, Malawi was one of four countries where MCP gains among the youngest women of reproductive age (15–19) were significant. Malawi also had the largest MCP gain among women with no education – at over 16% ^– [Bibr ref-22]^.

As the family planning community begins to look ahead to the FP2030 partnership, this analysis can help inform the emerging FP2030 family planning framework, which includes equity as a guiding principle ^23^. Helping family planning stakeholders understand whether the ambitious goal of the FP2020 partnership impacted equity will be critically important in determining how to establish goals and operationalize equity in the future family planning partnership. From our findings, we interpret the impact of the FP2020 partnership on equity to be positive or at a minimum neutral, and do not see signs of exacerbated inequality within countries. Countries engaged with the partnership through country-specific commitments, and those that set country-specific priorities to reduce inequitable access to modern contraceptive counseling and services, reaped the benefits.

While our analysis provides policy-relevant findings for the next family planning partnership, it had some limitations that should be noted. With our analysis confounding is likely and while regression approaches can mitigate against confounding, our aim was not to discern socio-demographic specific effect sizes or confirm associations. For example, our main objective was not to understand how different socio-demographic factors such as being wealthy versus poor increase the odds or probability of modern contraceptive use. Our primary objective was to understand if MCP significantly increased across different dimensions of inequities between two surveys, and a simple weighted bivariate analysis sufficed. Furthermore, with a simpler methodological approach (which is also more conservative in testing for significance), interpretability of findings is greater. Household surveys such as the MICS and DHS are not powered to detect significant changes among a relatively small portion of unmarried, married sexually active women; if changes among this sub-population of women are of special interest for family planning programming, other methods need to be employed to more precisely estimate their modern contraceptive use. Furthermore, in some countries the proportion of women in “no education” or “poorest” groups declined over time. It’s unclear whether this aided countries in providing family planning services to these groups. Our analyis does not address this.

This analysis addresses if FP2020’s overall “120 million additional users” goals led to the priorizitzation of easier to reach populations and exacterbated inequities; our findings can be used to inform the FP2030 partnership and reinforce that country-specific commitments, policies, and programming is likley to have the largest net effect on increasing or retaining modern contraceptive users and leaving no one behind.

## Data availability

### Underlying data

**DHS**

Data used in this study are from the HR and IR datasets of the Bangladesh 2014 and 2011 DHS; the HR and IR datasets of the Burundi 2016–17 and 2012 DHS; the HR and IR datasets of the Ethiopia 2016 and 2011 DHS; the HR and IR datasets of the Haiti 2016–17 and 2012 DHS; the HR and IR datasets of the Malawi 2015–16 and 2012 DHS; the HR and IR datasets of the Nepal 2016 and 2011 DHS; the HR and IR datasets of the Pakistan 2017–28 and 2012–23 DHS; the HR and IR datasets of the Senegal 2017 and 2012 DHS; the HR and IR datasets of the Uganda 2016 and 2011 DHS; and the HR and IR datasets of the 2015 and 2010–11 Zimbabwe DHS, available from the DHS website. Access to the dataset requires registration and is granted only for legitimate research purposes. A guide for how to apply for dataset access is available at: https://dhsprogram.com/data/Access-Instructions.cfm.

**MICS**

Data used in this study are from the households (HH) and women (WM) datasets of the Sierra Leone 2010, 2016, MICS, available from the MICS website. Access to the dataset requires registration and is granted only for legitimate research purposes. Questions about data access can be directed to mics@unicef.org.

### Extended data

Analysis code available from: https://github.com/familyplanning2020/NOLB/tree/v1.0

Archived analysis code at time of publication: https://doi.org/10.5281/zenodo.5075758 ^24^.

License: GNU General Public License.
