# Supplementary material for: No one left behind: has the pursuit of FP2020’s 120 million additional users goal left some women behind?
Source: Gates Open Res. 2022 Jan 28;5:114. Originally published 2021 Jul 28. [Version 2] doi: 10.12688/gatesopenres.13339.2 (PMC10409982; doi:10.12688/gatesopenres.13339.2)
Supplement: Peer review history

# Peer review: Reviewer response for version 2

**DOI:** 10.21956/gatesopenres.14777.r34477

**Published:** 2023-08-14

**Authors:** Ifunanya Clara Agu

**Affiliations:** 1 Health Policy Research Group, University of Nigeria, Enugu, Nigeria

This is a well-written report relating to family planning (FP2020). I appreciate the time and effort taken to produce this report. It was interesting and easy to read. I have two suggestions for the authors. 

 Although the discussion was well structured, I suggest that authors should review literature to check if there are related (global, regional, or national) reports/ articles with similar or conflicting findings for adequate comparison (mainly for the first, second, and third paragraph of the discussion section).

 Can authors also point out in the discussion section that in most advantaged groups (eg urban, richest) there was inconsistency / ‘no’ significant MCP gain in many countries? This is also a significant finding because many resources are channeled to disadvantaged groups with little focus on other population categories.

Is the work clearly and accurately presented and does it cite the current literature?

Yes

If applicable, is the statistical analysis and its interpretation appropriate?

I cannot comment. A qualified statistician is required.

Are all the source data underlying the results available to ensure full reproducibility?

Yes

Is the study design appropriate and is the work technically sound?

Yes

Are the conclusions drawn adequately supported by the results?

Partly

Are sufficient details of methods and analysis provided to allow replication by others?

Yes

Reviewer Expertise:

Health promotion, Sexual and reproductive health, Adolescent and young people's health, Family Planning

I confirm that I have read this submission and believe that I have an appropriate level of expertise to confirm that it is of an acceptable scientific standard.

# Peer review: Reviewer response for version 2

**DOI:** 10.21956/gatesopenres.14777.r33909

**Published:** 2023-08-08

**Authors:** Linnea A Zimmerman

**Affiliations:** 1 Department of Population, Family and Reproductive Health, Johns Hopkins Bloomberg School of Public Health, Baltimore, MD, USA

Thank you for the opportunity to review. My comments are largely minor and for the purpose of clarification. My most significant critique is in point 1 below, as it is not clear to me why authors did not explicitly show differences across most/least advantaged over time. Overall, however, the paper is well-written and contributes to relevant discussions related to equity. 

 1. While the authors do cite relevant literature, I think reference to the Choi and Fabic article "Monitoring Progress in Equality for the Sustainable Development Goals..." ^1^ noted below could help support introduction and discussion given their initial exploration of inequalities. I also think that Choi and Fabic's methodology (i.e. testing the differences between the most and least advantaged groups) could be replicated here. While authors show how MCP has changed across different groups, they don't actually show whether the difference between those groups has changed over time (though one can extrapolate). If you are not going to explicitly show how differences across groups has changed, at least justify why such an approach was not chosen.

 2. Regarding wealth categories, I was not clear on whether these quintiles were defined cross-country or within country. For example, if women were in the poorest households, were these the poorest within their own country or the poorest across all countries? I assume the latter but a slight clarification would make this more clear.

 2. Figure 1 - Rather than have two colors differentiating older/later surveys could authors differentiate each country with color/symbol combinations? I think this would make interpretation easier and faster.

 3. Figure 2- I applaud the authors creativity in finding a way to visualize quite a bit of information. On the whole I like the chart, but did find some sections where I could not find countries across time. For example Nepal/older in panel 3. If Nepal older is the turquoise dot near Malawi, what is the purple dot? Would the addition of shapes help? Defer to the authors but think a critical review is necessary to make sure the changes in all countries can be easily located in each panel. 

 4. Very minor change - suggest rewording "Increases were significant in seven countries– Burundi, Ethiopia, and Uganda in rural settings" to "Increases in rural settings were significant in seven countries– Burundi, Ethiopia, and Uganda"

 5. Is it possible to show change in education status, similar to what you did for wealth, to demonstrate both how mcp use has changed and how education status has changed? Fine if not, but it seems like it would compliment the wealth analysis.

Is the work clearly and accurately presented and does it cite the current literature?

Partly

If applicable, is the statistical analysis and its interpretation appropriate?

Yes

Are all the source data underlying the results available to ensure full reproducibility?

Yes

Is the study design appropriate and is the work technically sound?

Partly

Are the conclusions drawn adequately supported by the results?

Yes

Are sufficient details of methods and analysis provided to allow replication by others?

Yes

Reviewer Expertise:

Family planning, demography, survey analysis

I confirm that I have read this submission and believe that I have an appropriate level of expertise to confirm that it is of an acceptable scientific standard, however I have significant reservations, as outlined above.

## References

[1] : Monitoring Progress in Equality for the Sustainable Development Goals: A Case Study of Meeting Demand for Family Planning. *Glob Health Sci Pract* .2018;6(2) : 10.9745/GHSP-D-18-00012 390-401 10.9745/GHSP-D-18-00012 29959277PMC6024626

# Peer review: Reviewer response for version 2

**DOI:** 10.21956/gatesopenres.14777.r31695

**Published:** 2022-02-08

**Authors:** Shawn Malarcher

**Affiliations:** 1 United States Agency for International Development, Washington, DC, USA

Thanks for your careful consideration of my feedback. I found the graphics much improved.

 Please ensure that men are included when considering contraceptive choice.

Is the work clearly and accurately presented and does it cite the current literature?

Partly

If applicable, is the statistical analysis and its interpretation appropriate?

Partly

Are all the source data underlying the results available to ensure full reproducibility?

Yes

Is the study design appropriate and is the work technically sound?

Partly

Are the conclusions drawn adequately supported by the results?

Partly

Are sufficient details of methods and analysis provided to allow replication by others?

Yes

Reviewer Expertise:

family planning, social science research, KM, research translation

I confirm that I have read this submission and believe that I have an appropriate level of expertise to confirm that it is of an acceptable scientific standard.

# Peer review: Reviewer response for version 1

**DOI:** 10.21956/gatesopenres.14582.r31004

**Published:** 2021-08-26

**Authors:** Jamaica Corker

**Affiliations:** 1 The Bill & Melinda Gates Foundation, Seattle, WA, USA

This paper seeks to as answer several critical questions on the impact of FP2020’s goal of 120 million additional users (“120x20”), specifically whether certain groups of women were systematically left behind. I believe it makes an important contribution by providing an early indication that groups of women who are generally disadvantaged do not appear to have been left behind in FP2020 commitment countries.

 However, I think this is fundamentally different from questions around equity. As I see it, this paper’s contribution is not as the definitive answer to the elusive “equity” question in FP but rather to ascertain whether data from a select set of commitment countries indicates that progress under FP2020 exacerbated gaps in use between advantaged and disadvantaged groups. While it is difficult to draw many clear conclusions from this analysis (as nearly all results are inconsistent across countries), the results presented indicate that among FP2020 commitment countries there were not systematically greater MCP gains by the most advantaged women. This is a substantial finding that is almost buried under discussions about equity, programs and policies to which the data used does not directly speak. To strengthen this paper and to make the results and interpretation more robust, I suggest the authors stick more closely to the stated and important goal of ascertaining whether “increases in mCPR did not vary significantly in more disadvantaged groups” since the 2012 London Summit.

**Detailed feedback:**

1. I suggest the authors focus the scope of this analysis and interpretation more specifically on the contraceptive use (or demand satisfied – see point #3) outcomes they can directly measure with DHS and MICS data used here. Though the authors say “we examine FP2020 country data and policies for signs of inequity” the data and methods in this paper do not allow it to comment directly on the *focus of programs *or *policies*. Such an analysis would require more comprehensive (not just selective) data on programmatic and policy decisions, with adequate lag time for analysis. 
2. I question the appropriateness of MCP growth for this analysis. Without some underlying or contextual measure of demand or access, using MCP alone to measure equity implies that there is some ideal level of MCP across all populations – and the higher, the better (in contradiction to the FP2020 principles of agency and autonomy referenced). Could the authors look at changes in demand satisfied, arguably a more appropriate measure of equity given the different underlying levels of demand for contraception among population sub-groups? Flawed though unmet need is as a direct measure of demand for contraception, including it as a backdrop here (via demand satisfied) would better contextualize “equitable” FP changes.
  - If the authors choose to stick with MCP change, this should be more clearly addressed as an (acceptable) limitation. It would also be good to better contextualize demand/unmet need in the context of the FP2020 goal (i.e., the 120x20 goal was devised against the backdrop of more than 230 million women with unmet need) and in interpreting the results.
3. The paper is fundamentally asking about the impact of the FP2020 initiative, but there is no counterfactual or contextual information about what differential mCPR growth looked like prior to FP2020, nor a recognition of the limitation of comparing two-point estimates, especially among sometimes small sub-groups. FP2020 shifted our field to track progress using model-based estimates from FPET to overcome limitations of tracking changes between two surveys. Did the authors consider a modified FPET to see to what degree different sub-groups in countries followed or outperformed the pre-FP2020 trajectories that would have been expected?
4. The question of whether some women (e.g., the poor or those with no education) are systematically left behind is different from questions of equity. The question of how to define and measure equity in the context of FP programs is one the field continues to struggle with. I am not convinced that the authors’ definition of “equitable” gains as those that are equal to or larger among a more “disadvantaged” group suffices, as this implies an underlying goal of having consistent levels or contraceptive use across all sub-groups/dimensions of equity (regardless of demand). Likewise, is it “equitable” if any groups show slower growth or declines in MCP (as we see evidence from this analysis with some more advantaged groups)? And given starting differential in MCP across sub-groups in many countries, might we expect growth to be slower or faster depending on their placement along the s-curve? While this paper cannot answer those or all questions around the definition of equity in FP programming, it can be clearer about when it is commenting about MCP gains among groups that are generally disadvantaged with FP outcomes (e.g., the poor, less educated, youth) versus the more elusive concept of equity. 
5. The utility of using the International Wealth Index in place of the country-specific wealth measures (e.g., DHS quintiles) is not clear to me, given that countries are the unit of analysis. The shifts in population composition with the IWI measure seems more relevant for examining the relationship between absolute levels of wealth and contraceptive use or for understanding compositional influences on changes in MCP, but not necessarily for analyzing whether *within each country* women who are relatively poorer or wealthier appeared to be disadvantaged by policies related to the FP2020 goal. Though I find this analysis interesting, I don’t think it is necessary or that it strengthens the findings.
6. It appears that a handful of other long-term FP2020 commitment countries have DHS surveys that have two surveys between 2010 and 2019 were not included (Benin, Cameroon, Guinea and Mozambique, to name a few). If the inclusion criteria are more selective than mentioned (e.g., if one survey has to be prior to 2012), that should be specified. Alternatively, if some eligible countries were overlooked for this analysis, including them would make the findings more robust.
7. Is there a reason for using the Bangladesh 2014 DHS and not the 2017-18 survey? Unless there is a compelling reason related to the data, using the 2017-18 survey instead will provide a longer time lapse to better measure cumulative influence of FP2020.
8. The selective citing of policies related to increases in MCP among certain groups (e.g., Malawi’s focus on youth) distracts from the overall finding/message (that FP2020 commitments, as they were applied within countries, did not seem to exacerbate inequalities writ large). These policies are also cited inconsistently – e.g., credited for MCP gains among Malawi youth but no such policy is cited for the substantial gains among Malawi’s uneducated women – and thus come across as selective.
9. I generally found the graphics challenging to read and interpret, with the key messages not clearly represented visually. Perhaps fewer and simpler graphics could better convey the main findings.
10. I’d like more discussion about implications of the findings that some advantaged groups have experienced MCP stagnation or declines. Does this imply an inequitable focus on these groups, suggesting further declines among these groups under the FP2020 “equity” approach? Unlikely, but this could still have implications about how the FP2030 defines equity moving forward. After reinforcing their message that in its aim of reaching 120 million additional users FP2020 does not seem to have widened the use gap between advantaged and disadvantaged women, I’d encourage the authors to give a bit more consideration to their findings on MCP declines among several more advantaged groups and the implications this might have for FP2030. 

Is the work clearly and accurately presented and does it cite the current literature?

Yes

If applicable, is the statistical analysis and its interpretation appropriate?

Partly

Are all the source data underlying the results available to ensure full reproducibility?

Yes

Is the study design appropriate and is the work technically sound?

Partly

Are the conclusions drawn adequately supported by the results?

Partly

Are sufficient details of methods and analysis provided to allow replication by others?

Yes

Reviewer Expertise:

Fertility, family planning, demography

I confirm that I have read this submission and believe that I have an appropriate level of expertise to confirm that it is of an acceptable scientific standard, however I have significant reservations, as outlined above.

# Response

**Published:** 2021-12-21

**Authors:** Shiza Shiza

**Affiliations:** Avenir Health/FP2030, USA

Overall, we found your feedback to be incredibly helpful. Thank you for taking the time to make comments. Specific questions are addressed below. 

1. I suggest the authors focus the scope of this analysis and interpretation more specifically on the contraceptive use (or demand satisfied – see point #3) outcomes they can directly measure with DHS and MICS data used here. Though the authors say “we examine FP2020 country data and policies for signs of inequity” the data and methods in this paper do not allow it to comment directly on the *focus of programs *or *policies*. Such an analysis would require more comprehensive (not just selective) data on programmatic and policy decisions, with adequate lag time for analysis. 
  - We agree we would need additional data to discuss how program and policies impacted MCPR gains. Hence why it is not a focus of this paper and we removed any references to this. Thank you for the thorough check. 
2. I question the appropriateness of MCP growth for this analysis. Without some underlying or contextual measure of demand or access, using MCP alone to measure equity implies that there is some ideal level of MCP across all populations – and the higher, the better (in contradiction to the FP2020 principles of agency and autonomy referenced). Could the authors look at changes in demand satisfied, arguably a more appropriate measure of equity given the different underlying levels of demand for contraception among population sub-groups? Flawed though unmet need is as a direct measure of demand for contraception, including it as a backdrop here (via demand satisfied) would better contextualize “equitable” FP changes. If the authors choose to stick with MCP change, this should be more clearly addressed as an (acceptable) limitation. It would also be good to better contextualize demand/unmet need in the context of the FP2020 goal (i.e., the 120x20 goal was devised against the backdrop of more than 230 million women with unmet need) and in interpreting the results.
  - We opted to use MCPR because unmet need does not reliably follow a decreasing or increasing pattern. In fact, unmet need can increase in a country when demand for family planning is generated which would be difficult to quantify with population-based surveys like the DHS. Furthermore, demand satisfied is also a complex indicator which assumes that women’s demand is “satisfied” with the method that they are using. To avoid these issues, we opted to use a widely understood and used indicator of MCPR. This rationale was added in the method section. 
3. The paper is fundamentally asking about the impact of the FP2020 initiative, but there is no counterfactual or contextual information about what differential mCPR growth looked like prior to FP2020, nor a recognition of the limitation of comparing two-point estimates, especially among sometimes small sub-groups. FP2020 shifted our field to track progress using model-based estimates from FPET to overcome limitations of tracking changes between two surveys. Did the authors consider a modified FPET to see to what degree different sub-groups in countries followed or outperformed the pre-FP2020 trajectories that would have been expected?
  - We noted the limitation of not using regression approaches and using a simple bivariate analysis. On FPET, it is currently only set up to be used at the sub-national and national level. It cannot be tailored for different sub-populations in its current form.
4. The question of whether some women (e.g., the poor or those with no education) are systematically left behind is different from questions of equity. The question of how to define and measure equity in the context of FP programs is one the field continues to struggle with. I am not convinced that the authors’ definition of “equitable” gains as those that are equal to or larger among a more “disadvantaged” group suffices, as this implies an underlying goal of having consistent levels or contraceptive use across all sub-groups/dimensions of equity (regardless of demand). Likewise, is it “equitable” if any groups show slower growth or declines in MCP (as we see evidence from this analysis with some more advantaged groups)? And given starting differential in MCP across sub-groups in many countries, might we expect growth to be slower or faster depending on their placement along the s-curve? While this paper cannot answer those or all questions around the definition of equity in FP programming, it can be clearer about when it is commenting about MCP gains among groups that are generally disadvantaged with FP outcomes (e.g., the poor, less educated, youth) versus the more elusive concept of equity. 

- We discuss the issues of countries starting at different levels of MCPR in the discussion section.

1. The utility of using the International Wealth Index in place of the country-specific wealth measures (e.g., DHS quintiles) is not clear to me, given that countries are the unit of analysis. The shifts in population composition with the IWI measure seems more relevant for examining the relationship between absolute levels of wealth and contraceptive use or for understanding compositional influences on changes in MCP, but not necessarily for analyzing whether *within each country* women who are relatively poorer or wealthier appeared to be disadvantaged by policies related to the FP2020 goal. Though I find this analysis interesting, I don’t think it is necessary or that it strengthens the findings.
  - You cannot compare DHS or MICS wealth quintiles across time even within country. Since the wealth index is based on a principal components analysis for each survey, it makes it incomparable across time even in the same country.
2. It appears that a handful of other long-term FP2020 commitment countries have DHS surveys that have two surveys between 2010 and 2019 were not included (Benin, Cameroon, Guinea and Mozambique, to name a few). If the inclusion criteria are more selective than mentioned (e.g., if one survey has to be prior to 2012), that should be specified. Alternatively, if some eligible countries were overlooked for this analysis, including them would make the findings more robust.
  - We only included surveys that were released by June 2019.
3. Is there a reason for using the Bangladesh 2014 DHS and not the 2017-18 survey? Unless there is a compelling reason related to the data, using the 2017-18 survey instead will provide a longer time lapse to better measure cumulative influence of FP2020.
  - We only included surveys that were released by June 2019 and the Bangladesh 2017-18 had not been released by June 2019.
4. The selective citing of policies related to increases in MCP among certain groups (e.g., Malawi’s focus on youth) distracts from the overall finding/message (that FP2020 commitments, as they were applied within countries, did not seem to exacerbate inequalities writ large). These policies are also cited inconsistently – e.g., credited for MCP gains among Malawi youth but no such policy is cited for the substantial gains among Malawi’s uneducated women – and thus come across as selective.
  - On the first point, the overall focus of the paper is to assess if FP2020’s 120 million goal exacerbated inequities in countries. The focus of the paper is not on the country commitments themselves. We use the Malawi example to show that countries focused on implementing policies that supported reducing inequities.
  - On the second point, we cited that Malawi prioritized youth access to family planning in their National Friendly Health Services Strategy 2015-2020. The same policy also highlights the importance of education and prioritizing educational attainment among adolescent girls – we just did not cite this directly in the paper.
5. I generally found the graphics challenging to read and interpret, with the key messages not clearly represented visually. Perhaps fewer and simpler graphics could better convey the main findings.
  - We have added interpretation to the wealth and education graphics.
6. I’d like more discussion about implications of the findings that some advantaged groups have experienced MCP stagnation or declines. Does this imply an inequitable focus on these groups, suggesting further declines among these groups under the FP2020 “equity” approach? Unlikely, but this could still have implications about how the FP2030 defines equity moving forward. After reinforcing their message that in its aim of reaching 120 million additional users FP2020 does not seem to have widened the use gap between advantaged and disadvantaged women, I’d encourage the authors to give a bit more consideration to their findings on MCP declines among several more advantaged groups and the implications this might have for FP2030. 
  - The changes in modern contraceptive use among the most advantage groups (e.g. most education, richest, oldest, urban) were not significant. We have added this note into the discussion.

# Peer review: Reviewer response for version 1

**DOI:** 10.21956/gatesopenres.14582.r31003

**Published:** 2021-08-12

**Authors:** Shawn Malarcher

**Affiliations:** 1 United States Agency for International Development, Washington, DC, USA

The article analyzes survey data to examine the question of how well countries have performed in reducing inequities in FP. 

 My responses "partly" are due to areas where the analysis was unclear or difficult to follow (see comments below).

 I applaud the authors on this important review of FP programs. This is an important analysis and should help drive the next generation of support. While the authors reference important work in this area, it will be important to recognize explicitly a few key points:

1. While MCPR is a useful measure for this exercise, it does not provide the full picture of equity in FP. For example, unmet need for contraception may provide a better measure at how well programs are meeting demand. Likewise, MCPR does not capture client experience or the ability of the system to provide a preferred method. For purposes of this analysis, I agree MCPR works well, however, I suggest the authors acknowledge this limitation.
2. The current version is a high quality academic paper. It would be helpful to include some recognition that inequities are a result of the system's inability to serve the needs of particular communities. These differences mark important differences in power structures and resource allocation.
3. Following the point above, in countries where the equity gaps has shrunk, it would be helpful to acknowledge that this is due to the program's ability to shift these power and resource imbalances. They have been able to reshape programs to reflect the values and preferences in those communities where change has occurred. Systems are perfectly built to achieve the results they achieve (to change those results the system must change).
4. I have never seen MCP used. Is there a reason you aren’t using MCPR?
5. In the methods section the authors refer to “cross country analysis”, but I don’t see any cross country comparisons. Please clarify.
6. Overall, I have a difficult time with the graphics. The data is grouped by characteristic rather than by country so it is difficult to see what changes are happening in a country. The wealth graphic Figure 2 is very complex and challenging to read. Why does Burundi have 2 bubbles in the poorest column? I like Table 1. This one is easy to read. It would be more interesting to present the diff in diff analysis.
7. I appreciate the reference and cut off according to confidence interval, but is there a way to include them in the paper?  
8. I don’t believe it is appropriate to compare the most vulnerable (poorest) with the most advantaged (wealthiest). I recommend focusing your analysis on comparing the most vulnerable to an index population (poorest with middle income), as you have done and drop the other analysis. This comparison is more appropriate in my view and could make the figures and analysis easier to communicate. This is true for education and age as well.
9. The residential analysis doesn’t reflect on shifts in demographics. Was there a significant shift in people from rural to urban settings?
10. I can’t follow the finding on age. Please clarify. Did you limit the analysis to married women 15-19? Did you explore using sexually active 15-19 yrs olds?
11. The conclusion is a bit difficult to follow. It’s not just about – were there any significant gains in vulnerable populations. What we want to see is that programs focused on these groups and we saw them “catch up”. How did their gains compare with gains elsewhere? Perhaps you could start the conclusion with some general statements about which population groups saw the greatest increases in country (vulnerable or not) and then focus on the countries that show significant gains in reducing inequities.

Is the work clearly and accurately presented and does it cite the current literature?

Partly

If applicable, is the statistical analysis and its interpretation appropriate?

Partly

Are all the source data underlying the results available to ensure full reproducibility?

Yes

Is the study design appropriate and is the work technically sound?

Partly

Are the conclusions drawn adequately supported by the results?

Partly

Are sufficient details of methods and analysis provided to allow replication by others?

Yes

Reviewer Expertise:

family planning, social science research, KM, research translation

I confirm that I have read this submission and believe that I have an appropriate level of expertise to confirm that it is of an acceptable scientific standard, however I have significant reservations, as outlined above.

# Response

**Published:** 2021-12-21

**Authors:** Shiza Shiza

**Affiliations:** Avenir Health/FP2030, USA

Overall, we found your feedback to be incredibly helpful. Thank you for taking the time to make comments. Specific questions are addressed below.

1. While MCPR is a useful measure for this exercise, it does not provide the full picture of equity in FP. For example, unmet need for contraception may provide a better measure at how well programs are meeting demand. Likewise, MCPR does not capture client experience or the ability of the system to provide a preferred method. For purposes of this analysis, I agree MCPR works well, however, I suggest the authors acknowledge this limitation.
  - We added additional information on this in the methods section where we discuss why we opted to use MCPR and then also added in the discussion section limitation of how our analysis in unable to provide an analysis on client experience or the ability of the system to provide a woman’s preferred method.  
2. The current version is a high quality academic paper. It would be helpful to include some recognition that inequities are a result of the system's inability to serve the needs of particular communities. These differences mark important differences in power structures and resource allocation.
  - We added in a note on how resource allocation and tailored programming towards reducing inequities is likely what allowed countries to grow MCPR. 
3. Following the point above, in countries where the equity gaps has shrunk, it would be helpful to acknowledge that this is due to the program's ability to shift these power and resource imbalances. They have been able to reshape programs to reflect the values and preferences in those communities where change has occurred. Systems are perfectly built to achieve the results they achieve (to change those results the system must change).
  - We did not measure the impact of programs or resource allocation therefore we cannot assume that is what led to inequities shrinking. We did add a note about how resource allocation towards equity focused programs likely supported equitable growth in MCPR.
4. I have never seen MCP used. Is there a reason you aren’t using MCPR?
  - We edited MCP to MCPR.
5. In the methods section the authors refer to “cross country analysis”, but I don’t see any cross country comparisons. Please clarify.
  - What was meant by cross country analysis is that you can compare change over time and across different countries.
6. Overall, I have a difficult time with the graphics. The data is grouped by characteristic rather than by country so it is difficult to see what changes are happening in a country. The wealth graphic Figure 2 is very complex and challenging to read. Why does Burundi have 2 bubbles in the poorest column? I like Table 1. This one is easy to read. It would be more interesting to present the diff in diff analysis.
  - All countries in the wealth graphic should have two bubbles. One bubble is for the earlier survey and the second bubble is for the later survey. We opted not to do a regression analysis (which would be a difference in difference analysis) and our reasoning added to the discussion section. We will add additional notes that will help with the interpretation.
7. I appreciate the reference and cut off according to confidence interval, but is there a way to include them in the paper?  
  - The confidence intervals are included in the paper. For interpretability of the findings, we opted to keep them in tables and not directly in text.
8. I don’t believe it is appropriate to compare the most vulnerable (poorest) with the most advantaged (wealthiest). I recommend focusing your analysis on comparing the most vulnerable to an index population (poorest with middle income), as you have done and drop the other analysis. This comparison is more appropriate in my view and could make the figures and analysis easier to communicate. This is true for education and age as well.
  - We wanted to compare the most disadvantaged group to the least disadvantaged group because we wanted to assess if the most vulnerable (disadvantaged) were left behind while the most advantaged continued to progress. While we focus on these two groups in most of the analysis, we do calculate MCPR among all levels of the equity dimensions and those comparisons can be made by all readers by reviewing the tables.  
9. The residential analysis doesn’t reflect on shifts in demographics. Was there a significant shift in people from rural to urban settings?
  - We investigated this question, and we did not see any change in people moving from rural to urban settings in every country between surveys.
10. I can’t follow the finding on age. Please clarify. Did you limit the analysis to married women 15-19? Did you explore using sexually active 15-19 yrs olds?       
  - For the age analysis, the findings are for all women where possible. There is a separate analysis for unmarried sexually active women.
11. The conclusion is a bit difficult to follow. It’s not just about – were there any significant gains in vulnerable populations. What we want to see is that programs focused on these groups and we saw them “catch up”. How did their gains compare with gains elsewhere? Perhaps you could start the conclusion with some general statements about which population groups saw the greatest increases in country (vulnerable or not) and then focus on the countries that show significant gains in reducing inequities.
  - We do start the conclusions with some general statements. Since we cannot measure direct program effect in this paper, we added a point in the discussion where we mention this has a limitation.
